# Inhibiting the Unconventionals: Importance of Immune Checkpoint Receptors in γδ T, MAIT, and NKT Cells

**DOI:** 10.3390/cancers13184647

**Published:** 2021-09-16

**Authors:** Elisa Catafal-Tardos, Maria Virginia Baglioni, Vasileios Bekiaris

**Affiliations:** Section of Experimental and Translational Immunology, Department of Health Technology, Technical University of Denmark, Kemitorvet, Bldg 202, 2800 Kgs Lyngby, Denmark; elcat@dtu.dk (E.C.-T.); vikybaglioni@gmail.com (M.V.B.)

**Keywords:** immune checkpoint receptors, cancer, immunotherapy, γδ T cells, MAIT cells, NKT cells

## Abstract

**Simple Summary:**

All conventional major histocompatibility complex (MHC)-restricted T cells transiently express immune checkpoint/inhibitory receptors (ICRs) following activation as a means to counter-regulate overactivation. However, tumors promote chronic ICR expression rendering T cells chronically unresponsive or “exhausted”. Checkpoint inhibitor (CPI) therapy targets and blocks ICRs, restoring T cell activation and anti-tumor immunity. However, CPI therapy often fails, partly because of the tumor’s many abilities to inhibit MHC-driven T cell responses. In this regard, our immune system contains an arsenal of unconventional non-MHC-restricted T cells, whose importance in anti-tumor immunity is rapidly gaining momentum. There is currently little knowledge as to whether unconventional T cells can get exhausted and how CPI therapy affects them. In this article we review the current understanding of the role of ICRs in unconventional T cell biology and discuss the importance of targeting these unique immune cell populations for CPI therapy.

**Abstract:**

In recent years, checkpoint inhibitor (CPI) therapy has shown promising clinical responses across a broad range of cancers. However, many patients remain unresponsive and there is need for improvement. CPI therapy relies on antibody-mediated neutralization of immune inhibitory or checkpoint receptors (ICRs) that constitutively suppress leukocytes. In this regard, the clinical outcome of CPI therapy has primarily been attributed to modulating classical MHC-restricted αβ T cell responses, yet, it will inevitably target most lymphoid (and many myeloid) populations. As such, unconventional non-MHC-restricted gamma delta (γδ) T, mucosal associated invariant T (MAIT) and natural killer T (NKT) cells express ICRs at steady-state and after activation and may thus be affected by CPI therapies. To which extent, however, remains unclear. These unconventional T cells are polyfunctional innate-like lymphocytes that play a key role in tumor immune surveillance and have a plethora of protective and pathogenic immune responses. The robust anti-tumor potential of γδ T, MAIT, and NKT cells has been established in a variety of preclinical cancer models and in clinical reports. In contrast, recent studies have documented a pro-tumor effect of innate-like T cell subsets that secrete pro-inflammatory cytokines. Consequently, understanding the mechanisms that regulate such T cells and their response to CPI is critical in designing effective cancer immunotherapies that favor anti-tumor immunity. In this Review, we will discuss the current understanding regarding the role of immune checkpoint regulation in γδ T, MAIT, and NKT cells and its importance in anti-cancer immunity.

## 1. Introduction

Although the term “T lymphocyte” is synonymous with adaptive immune responses generated by B and CD4^+^ or CD8^+^ alpha beta (αβ) T cells, over the last 20 years or so, an increasing number of T cell populations have been proven to have innate-like properties. The unconventional innate-like T cells can be αβ or gamma delta (γδ) in origin with either invariant or diverse T cell receptors (TCR), which often are not major histocompatibility complex (MHC)-restricted and recognize non-peptide antigens either via direct interactions or in the context of non-polymorphic antigen-presenting molecules, such as CD1 and MR1 [1]. Innate TCRs thus have been shown to interact with lipids, glycolipids, vitamin derivatives, phosphoantigens, butyrophilins, and stress ligands to name a few, inducing an immediate immune response [1,2,3,4,5]. Such TCR-antigen interactions are reminiscent of how innate immune cells recognize pathogen and danger associated molecular patterns (PAMPs and DAMPs). In addition to their unique TCRs, all innate-like T cell subsets can respond directly to cytokines independent of antigen or TCR engagement, exemplifying their non-adaptive immune role [5,6,7]. The most well-studied unconventional T cell subsets include γδ T cells, mucosal associated invariant αβ T cells (MAIT) and natural killer αβ T cells (NKT). Innate-like T cells and their TCRs are conserved in evolution, appearing in bony and cartilaginous fish and amphibians [8]. Jawless fish, such as lamprey, have T-like cells that resemble both αβ and γδ T cells [9], indicating that diversification of innate-like T cells occurred before the emergence of recombinase activating genes and adaptive immunity. Thus, we have at our disposal a unique army of T cells, whose function evolved over millions of years to be rapid and targeted at the same time.

However, unchecked cellular activation and proliferation come at a price, often in the form of tissue damage, which may perpetuate as a chronic inflammatory disease. One of the mechanisms that immune cells have evolved in order to counteract their own efficiency is the expression of inhibitory, or immune checkpoint receptors (ICRs) as most commonly referred to. Most of what we know about the immunological importance of ICRs stems from work done in conventional CD4^+^ and CD8^+^ T cells, while detailed biological insight regarding innate leukocytes or unconventional T cells is not as extensive. However, recent data suggest a critical role of the ICR PD-1 (programmed death-1) in myeloid cells [10,11], while evidence from mouse and human studies supports a potential important role for different ICRs in innate-like T cell subsets.

After a brief overview on basic ICR biology and exhaustion, we will discuss why we think studying ICR biology in unconventional innate-like lymphocytes would be clinically beneficial. We will later summarize the current state-of-the-art regarding the role of ICRs in innate-like T cell biology. We would like to emphasize that upon researching the literature for this review article we discovered that there are surprisingly few studies investigating the importance of ICRs in unconventional T cells. We have therefore selected to discuss ICRs for which there was substantial information on their potential role MAIT, NKT, or γδ T cells.

## 2. Immune Checkpoint Receptors and the Path to Exhaustion

Most of the well-studied ICRs, such as PD-1 (programmed cell death-1), CTLA-4 (cytotoxic T lymphocyte antigen-4) or BTLA (B and T lymphocyte attenuator) are members of the immunoglobulin superfamily (IgSF); however, some can be C-type lectin-like receptors [12]. They are expressed on the surface of cells either constitutively (e.g., BTLA) or are induced soon after activation (e.g., PD-1) and in T cells often prevent overt proliferation and cytokine production [12,13,14]. At the molecular level, the majority of ICRs contain intracellular immunoreceptor tyrosine inhibitory or switch motifs (ITIM or ITSM), which can recruit the Src-homology-2 phosphatases 1 and 2 (SHP-1 and SHP-2) [12,15]. Upon ligand binding, the ITIM/ITSM motif will be phosphorylated, resulting in the recruitment and activation of SHP-1 and SHP-2, which in turn will initiate a cascade of de-phosphorylation events, counteracting therefore ongoing molecular events that promote cellular activation [12,14]. In addition to direct signaling, ICRs can inhibit cell activation by competing for ligands that otherwise bind activating receptors [12]. This is the main pathway by which CTLA-4 inhibits T cell activation. Hence, CTLA-4 binds with high affinity the B7 ligands CD80 and CD86 on the surface of antigen presenting cells and can thus outcompete CD28 [16]. The CD28-CD80/86 interaction is critical for T cell co-stimulation and its disruption by CTLA-4 shuts down T cell mediated responses. A more intricate inhibitory function has been shown for BTLA, whose only ligand is the tumor necrosis factor (TNF) superfamily receptor HVEM (herpes virus entry mediator) [17]. *Trans* binding of HVEM induces SHP1/2-mediated signaling downstream of BTLA [17]. However, BTLA and HVEM are co-expressed on T cells and can exist in a *cis*-complex, which restricts HVEM from binding its other ligands CD160 and LIGHT, suppressing thus HVEM-driven activation of the canonical nuclear factor kappa-B (NF-κB) pathway [18,19,20].

**Figure 1:**
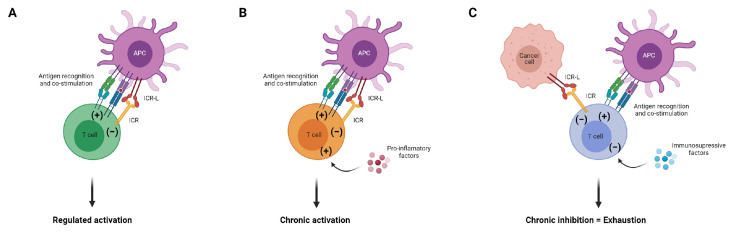
Balance between T cell activation and T cell inhibition. (**A**) Under normal conditions, the inhibitory signals mediated by ICR ligation down-regulate T cell responses to prevent immunopathology and autoimmunity. (**B**) During chronic inflammation, T cells are overwhelmed by several activation signals, overcoming ICR inhibition and tipping the balance towards constitutive T cell activation. (**C**) Despite the presence of antigen, in chronic viral infection and cancer, the immunosuppressive factors in the microenvironment break the equilibrium towards chronic T cell inhibition or exhaustion. APC: antigen-presenting cell; ICR: immune checkpoint receptor; ICR−L: immune checkpoint receptor ligand.

Since expression of ICRs and their ligands is driven by stimulation, inhibitory signals will persist for as long as the antigen is around and there is ongoing immune activation. This creates an equilibrium between T cell activation and T cell inhibition, allowing a regulated response and avoiding potential autoimmune reactions (Figure 1A). However, during chronic antigen persistence, the balance between activation and inhibition may break depending on the nature of the antigenic stimulus and the microenvironment where the T cell is recruited to. Hence, in autoimmune diseases, chronic antigenic stimulation and a microenvironment overwhelmed by pro-inflammatory cytokines favors chronic activation, leading to T cell mediated tissue destruction (Figure 1B). In view of this, despite constitutive PD-1 expression, T cells in idiopathic juvenile arthritis are active and pathogenic [21]. In contrast, in chronic viral infections and in many solid cancers, the persistent antigen is accompanied by immunosuppressive factors such as type I interferons (IFN), interleukin(IL)-10 and transforming growth factor-β1 (TGF-β1), shifting the balance towards inhibition [22] (Figure 1C). Consequently, the T cell is overcome by ICR-mediated negative signals, leading to its functional exhaustion and inability to fight transformed or infected cells.

## 3. Checkpoint Inhibitor Therapy: From Conventional to Unconventional

**Figure 2:**
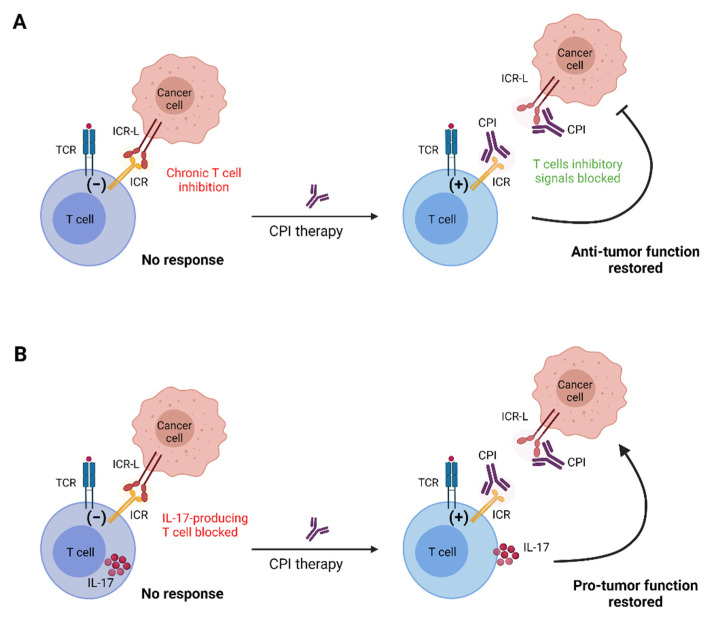
Checkpoint inhibition therapy. (**A**) ICR signaling in the tumor microenvironment inhibits T cell responses, thus contributing to tumor immune escape; CPI administration blocks ICR/ICR−L interactions, restoring T cell antitumor responses. (**B**) ICR-mediated inhibition of IL−17-producing T cells blocks their pro-tumor activity; CPI therapy targeting these cells will promote tumor growth. CPI: checkpoint inhibitor; ICR: immune checkpoint receptor; ICR−L: immune checkpoint receptor ligand; IL−17: interleukin 17; TCR: T cell receptor.

Reversing T cell exhaustion in cancer is the goal of checkpoint inhibitor (CPI) therapy. CPIs are monoclonal antibodies, which are designed to block ICRs on the surface of exhausted CD8^+^ T cells, releasing them thus from chronic inhibition, and restoring anti-tumor functionality [23] (Figure 2A). This therapeutic strategy has become a gold standard for many immunotherapies and for some cancers, such as melanoma, and may result in prolonged survival (2–3 years) for approximately 20–30% of patients [24]. Thus far only CPIs that target the PD-1 and CTLA-4 pathways have been approved for clinical use and include the anti-CTLA4 antibody ipilimumab, the anti-PD-1 antibodies nivolumab, pembrolizumab, cemiplimab, and the anti-PDL-1 antibodies atezolizumab, avelumab, and durvalumab [25].

**Figure 3:**
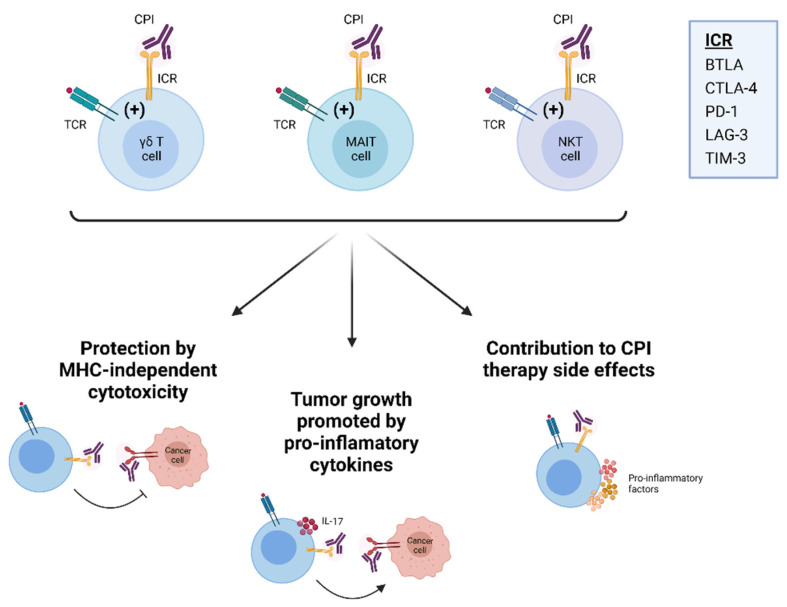
Potential importance of unconventional T cells during CPI therapy. Targeting γδ T cells, MAIT cells and NKT cells with CPI therapy may lead to strong MHC-independent anti-tumor responses. However, this treatment could also trigger the production of pro-inflammatory cytokines that promote tumor growth. In addition, unconventional T cell responses might contribute to the side effects often seen during CPI therapy. Abbreviations as in text.

Despite the frequently positive clinical results, many patients do not respond to CPI therapy [26], illustrating the need to better understand the underlying cellular and molecular mechanisms that lead to T cell exhaustion in order to improve efficacy. In this regard, resistance to CPI therapy is most often associated with impaired generation of tumor-specific primary and memory CD8^+^ T cells [27]. This is owing to the numerous ways by which the tumor microenvironment (TME) can suppress MHC-restricted antigen-driven immunity, and substantial efforts are underway in order to overcome this [27,28]. Most of these efforts are directed towards improving antigen recognition by CD8^+^ T cells [27,28]. However, in order to win the race against cancer, it is our view that efforts to overcome resistance to immunotherapy should expand in immune cell populations other than conventional T cells, especially given the fact that ICRs are expressed by most leukocytes. Excellent work for example demonstrated that the targeting of monocytes and macrophages could restore successful anti-tumor immunity [10,11,29]. We reason that it will be beneficial for CPI therapies to begin targeting non-MHC restricted anti-tumor T cells, such as γδ T, NKT, or MAIT cells. In this regard, it is critical to consider that innate-like T cell subsets have potent IL-17-producing capacities, which support tumor growth (see review by Paget and colleagues and Neubauer and colleagues in this issue). We and others have shown before that lack of ICR signaling can promote IL-17-driven γδ T cell immunity [30,31] and that pro-inflammatory cytokines can induce the expression of various ICRs on the surface of γδ T cells [32]. Furthermore, CPI therapy is frequently associated with adverse effects resembling various autoimmune disorders [33]. Given the potential rapid innate activation of unconventional T cells by ICR blockade, it is plausible that these cell subsets are actively involved in mediating such adverse effects. It will therefore be important to avoid that CPI therapy unleashes IL-17-producing T cells, which will favor tumor growth (Figure 2B) or induce side effects (Figure 3). To achieve this, it is important that we study and understand the biology of different ICRs in unconventional innate-like T cells.

## 4. The Three “Unconventionals”: γδ T, NKT and MAIT Cells

Of the unconventional trio, γδ T cells were the first ones discovered, and being the first T cell subset expressing non-α and non-β TCR variable genes, automatically made them the original member of the “unconventionals”. There is strong evidence from mouse and human that γδ T cell subsets with innate and innate-like function are pre-programmed in the thymus [34,35,36,37]. Interestingly, at least in the mouse, such innate subsets appear to have hyporesponsive TCR [38], despite the requirement for TCR signaling during their development [39,40]. Instead of recognizing antigen (Ag), cell activation is achieved through responses to cytokines, similar to innate lymphoid cells [41]. However, human γδ TCRs have been shown to recognize diverse non-peptide ligands, such as the B7-like molecules butyrophilins [2,4,42,43], MR1 [3], or annexin A2 [44]. γδ T cells can play key roles in many cancers [45,46,47], while their therapeutic potential in adoptive cell transfer immunotherapies has been recently demonstrated in pre-clinical models and clinical trials with varying success [47,48,49,50,51]. Furthermore, there is evidence for γδ T cell memory [52,53], indicating their importance in conferring both short- and long-term protection against cancer or infection.

Natural killer T (NKT) cells are αβ T cells that recognize lipid molecules in the context of CD1d presentation and can have invariant or diverse TCRs. Invariant or type I NKT cells were first described to react to the marine sponge derived α-galactosylceramide (α-GalCer) and since, a number of bacterial derived glycolipids have been identified as type I NKT antigens, demonstrating their unequivocal role in protective immunity and cancer [54]. Although antigen recognition leads to rapid activation and cytokine production, the type of response varies depending on the NKT cell subset and can be of type 1 (IFN-γ-secreting), type 2 (IL-4/13-secreting), or type 3 (IL-17-secreting) [5]. NKT cells with a more diverse TCR that do not react to α-GalCer are known as type II, and similar to their type I counterparts they too display functional diversity [54]. As expected, NKT cells have been found to promote immunity against pathogens and cancer, but to also be pathogenic in various inflammatory settings. In this regard, a number of clinical trials have investigated the potential of NKT cells in cancer immunotherapy (reviewed by Godfrey et al. [54]).

Similar to NKT, MAIT cells rearrange invariant α and biased β TCRs, however, they display reactivity to the non-polymorphic, MHC-like molecule MR1, which presents vitamin B metabolites [1]. Their exact role in both humans and animals is still not fully determined, however, they are believed to be critical for anti-bacterial responses. As the name suggests, MAIT cells are located in the mucosae, however, they can be found in blood and secondary lymphoid organs [1,54]. As is the case with their other unconventional partners, MAIT cells have recently been shown to have a strong association with anti-tumor responses, and in this regard, we would like to refer you to two excellent reviews by O’Neill et al. and Cogswell et al. in this issue [55,56].

## 5. Immune Checkpoint Receptor Inhibition in Unconventional T Cells

In the remaining sections we will discuss the importance of the ICRs BTLA, CTLA-4, PD-1, LAG-3, and TIM-3 (Table 1) in regulating unconventional T cell responses.

## 6. BTLA

BTLA is constitutively expressed by most lymphocytes and is the only ICR with a TNF receptor superfamily ligand, HVEM [19]. As mentioned above it can inhibit both by direct SHP-mediated signaling but also by preventing HVEM induction of NF-κB. It can inhibit B, T and dendritic cells (DCs) and seems to play an intricate role in regulating anti-tumor responses [57] with a very prominent role in follicular lymphomas [58]. In mouse γδ T cells, BTLA is expressed by both IL-17- and IFN-γ-producing subsets. Its expression is repressed by the transcription factor RORγt and as a result IL-17-producing γδ T cells (γδT17) as well as other RORγt-expressing lymphocytes have very low levels of surface BTLA [30]. Its expression however can be induced by cytokine activation, including IL-7, IL-23, and IL-1β [32]. Despite its low levels, mice deficient in BTLA have increased numbers of γδT17 cells that are hyperactive and more pathogenic in the context of skin inflammation [30]. In humans, BTLA has been studied in Vγ9Vδ2 cells. It was shown that through interactions with HVEM, BTLA could suppress Vγ9Vδ2 cell proliferation, most likely by attenuating TCR signaling [59]. Importantly, BTLA was highly expressed by Vδ2^+^ cells in the lymph nodes of patients with lymphoma, and could suppress their proliferation upon ligation by HVEM on primary tumors [59]. The importance of BTLA in suppressing human γδ T cell proliferation was recently confirmed [60]. The role of BTLA in MAIT cells or human NKT cells has not been investigated. Mice deficient in BTLA, however, are more susceptible to Con-A induced hepatitis due to hyperactive type I NKT cells [61,62]. In these mice, NKT cells produced higher amounts of cytokine in response to α-GalCer stimulation [61,62], suggesting that BTLA may be regulating the strength of signaling downstream of the TCR. In a mammary tumor mouse model, Weigert and colleagues showed that intratumoral type I NKT cells express high levels of BTLA, which is required to suppress their anti-cancer activity [63].

## 7. CTLA-4

CTLA-4 was one of the first ICRs to be cloned and early studies into its function established that it tightly regulates B7-CD28 mediated T cell co-stimulation [64,65]. The lymphoproliferative disorders and multi-tissue damage in CTLA-4 deficient mice affirmed the idea that T cell inhibition is a critical immunological function [66,67,68]. It was later shown that blockade of CTLA-4 with monoclonal antibodies could restore anti-tumor responses in mice [69], establishing the foundations for CPI therapy. Similar to BTLA, CTLA-4 can transmit inhibitory signals either through SHP1/2 or by antagonizing by binding with activating receptors, in this case CD28. Despite the overwhelming insight on the biology of CTLA-4 in conventional CD4^+^ and CD8^+^ T cells, and its successful targeting in cancer, we know remarkably little about how CTLA-4 may be regulating innate-like T cell responses in either mouse or human (e.g., we could not find any substantial study correlating CTLA-4 and NKT function).

In the context of infection, *Plasmodium vivax* infected individuals have exhausted γδ T cells with characteristically high levels of CTLA-4 among other ICRs [70], however, its contribution is undefined. A study that collected patient samples during the 2014–2015 Ebola virus outbreak, showed that infection led to very low numbers of blood Vδ2^+^ cells, and that patients who survived had lower levels of surface CTLA-4 on their Vδ2^+^ cells [71]. In melanoma, patients with decreased frequencies of Vδ2^+^ cells, had reduced overall survival upon treatment with ipilimumab, the CTLA-4 antagonist [72]. Although only correlative, these studies pinpoint towards a suppressive role of CTLA-4 in γδ T cells. Interestingly, CD86-expressing Vδ2^+^ cells could suppress αβ T cells by engaging CTLA-4 [73]. In a transplantation mouse model, CTLA-4 synergized with NKG2D to suppress γδT17 cells and prolong cardiac allografts [74].

CTLA-4 was found to be highly expressed in liver resident and blood MAIT cells from patients with autoimmune liver disease [75]. Besides CLTA-4, these patients’ MAIT cells expressed classic markers of exhaustion and displayed reduced capacity for IFN-γ production, paradoxically, however, secretion of MAIT-associated IL-17 was enhanced [75]. Similarly, MAIT cells from individuals with chronic hepatitis B infection expressed high levels of CTLA-4, together with PD-1, and were impaired in producing IFN-γ and granzyme B [76]. In line with this data, intratumoral MAIT cells of a cohort of hepatocellular carcinoma patients, co-expressed high levels of both CTLA-4 and PD-1, which correlated with mild exhaustion. However, whether MAIT-expressed CTLA-4 is directly or indirectly implicated in any of these diseases is currently not known. Recent transcriptional analyses showed that by comparison to blood, oral mucosa resident MAIT cells expressed very high levels of *CTLA4* [77]. In vitro stimulation experiments additionally suggested that cytokines alone, without the need for TCR engagement, are sufficient to induce robust surface CTLA-4 in MAIT cells [77].

## 8. PD-1

PD-1 is an IgSF ICR, first identified as a T cell receptor in 1992 [78], which interacts with two IgSF ligands, PDL-1, and PDL-2. While PDL-1 shows ubiquitous expression [79], PDL-2 is mainly expressed by innate immune cells [80]. Upon ligand binding, PD-1 initiates its inhibitory function via ITIM/ITSM-dependent recruitment of SHP1/2 [81,82]. The importance of PD-1 and its ligands in the immune system are exemplified by the six, thus far, FDA approved anti-PD-1/PDL blocking antibodies that are used for cancer therapy [25]. Although PD-1 is expressed by unconventional T cells, its role and relevance in these populations is underexplored.

In vitro studies with γδ T cells showed that similarly to conventional αβ T cells, Vδ2 cells upregulate PD-1 shortly after antigenic stimulation [83]. In adult Vδ2 cells, the expression of PD-1 peaks between 2 to 4 days after TCR activation, and subsequently declines gradually to moderate levels [83,84]. Neonatal Vδ2 cells, on the other hand, maintain high expression of PD-1 on their surface for longer periods of time following TCR stimulation [85]. Both cytotoxicity and IFN-γ production of in vitro activated PD-1^+^ Vδ2 cells could be inhibited following PDL-1 ligation [83]. In the context of cancer, high PD-1 expression has been reported on γδ T cells isolated from a variety of tumors, including metastatic neuroblastoma [86], colorectal cancer [87], and multiple myeloma [88]. In this regard, Vγ9Vδ2 cells isolated from the bone marrow of multiple myeloma patients showed higher levels of PD-1 expression compared to Vγ9Vδ2 cells derived either from blood or control bone marrow [88]. Furthermore, these PD-1^+^ γδ T cells showed impaired proliferative capacity upon antigen stimulation, which was partially restored by blocking PD-1 signaling [88]. Interestingly, a recent study revealed that PD-1 blockade enhances antibody-dependent cellular cytotoxicity (ADCC) of follicular lymphoma cells by CD16^+^ Vγ9 lymphocytes in an in vitro culture system [89]. In contrast to the previous studies, a recent report found no effect of PD-1 blockade in the cytotoxic activity of human γδ T cells towards leukemia cell lines [84]. PD-1 inhibition did increase IFN-γ production by γδ T cells after zoledronate (Zol) stimulation and after challenge with Zol-treated THP-1 cells as well as Zol-sensitized acute myeloid leukemia blasts, but there was no significant difference in the proliferation of these cells, or the expression of CD107a on their surface [84]. A meta-analysis comparing single-cell RNA-sequencing (scRNAseq) datasets from melanoma patients that responded or not to anti-PD-1 therapy, showed that there was a population of γδ T cells that its presence in the tumor correlated with non-responders [90]. However, further investigation into this γδ T cell subset (e.g., cytokine profiling) is missing.

Despite evidence that PD-1 can inhibit γδ T cell function and thus may alter protective anti-tumor responses, mouse studies have shown that PD-1 can also modulate γδT17 cells, which, as mentioned above, can promote tumor growth. As such, PD-1-deficient mice showed elevated numbers of γδT17 cells and were more susceptible to γδT17-driven skin inflammation [31], while activation of PD-1 signaling by PDL-1-Fc suppressed the production of IL-17A by γδ T cells and reduced psoriatic inflammation [91]. The possibility of adverse effects following anti-PD-1 therapy through γδT17 over-activation was recently shown in a mouse model of combination therapy. Acute radiation-induced lung injury was worsened in mice that received anti-PD-1 monoclonal antibodies, due to the increased production of IL-17A by γδ T cells [92]. Therefore, deepening our understanding of ICR regulation on not only bulk but also functionally distinct subsets of γδ T cells will be critical for designing efficacious PD-1-related combination therapies with minimal side effects.

PD-1 expression is also upregulated on invariant NKT cells following antigenic stimulation [93,94]. Early reports showed that PD-1^+^ NKT cells had an impaired capacity to produce IFN-γ, IL-4, and IL-12 after α-GalCer stimulation [93,95,96]. PD-1 blockade was able to restore NKT cell proliferation and cytokine production, leading to effective anti-tumor responses in a model of melanoma [93,97]. In contrast, a different study observed little impact of anti-PD1 treatment in rescuing anergic NKT cells [98]. In line with this, PD-1 blockade could prevent the development of dysfunctional NKT cells when administered at the same time as primary α-GalCer stimulation, but the treatment was not able to restore cytokine production once anergy had been established [95]. Thus, the beneficial effects of PD-1/PDL-1 blocking agents on type I NKT cells seem to be most pronounced when given at the time of antigen stimulation [99]. The therapeutic potential of α-GalCer/anti-PD-1 combination therapy was recently evaluated in a pre-clinical model of colorectal cancer. In this setting, the individual administration of α-GalCer or anti-PD-1 had very limited effect on cancer progression [100]. The combination of both therapies, however, resulted in increased activation and proliferation of NKT cells in the tissues, and strongly suppressed the development of polyps in both small intestine and colon [100]. In addition, α-GalCer/anti-PD-1 significantly increased PLZF^+^Tbet^+^ NKT cells in the polyps over other invariant NKT cell subtypes [100], suggesting that PD-1 regulation of NKT cells could be subset-dependent. The regulatory role of PD-1 on NKT cells is not limited to murine models [94,101]. NKT cells obtained from non-small cell lung cancer patients show increased expression of PD-1 and reduced proliferation capacity compared with healthy controls [94]. α-GalCer stimulation induced PD-1 expression on human NKT cells, which inhibited cytokine production [94]. In addition, PDL-1 blockade increased the cytotoxicity of NKT cells against several tumor cells lines [94]. Thus, PD-1 inhibition seems to influence NKT cell responses in human cancers.

PD-1 expression during chronic inflammation has also been associated with functional impairment of MAIT cells [76,102,103]. Moreover, PD-1 blockade was able to restore cytokine production in dysfunctional MAIT cells derived from active tuberculosis patients [102]. In the context of cancer, elevated levels of PD-1 have been reported on MAIT cells derived from hepatocellular carcinoma, esophageal adenocarcinoma, and colorectal cancer patients [104,105,106]. In the latter study, PD-1^+^ MAIT cells that co-expressed TIM-3 showed increased proliferative capacity and diminished polyfunctionality compared to their PD-1^−^TIM-3^−^ counterparts [106]. In addition, a recent report compared MAIT gene expression profiles in paired samples from cancer patients before and after anti-PD-1 therapy. PD-1 blockade increased the expression of activation genes in MAIT cells derived from basal and squamous cell carcinoma patients, suggesting a functional role of PD-1 in the regulation of this cell type [107]. Moreover, scRNAseq profiling of intratumoral lymphocytes of metastatic melanoma patients revealed that highly active MAIT cells can correlate with better prognosis to PD-1 blockade [108].

## 9. LAG-3

Lymphocyte activation gene-3 (LAG-3), also known as CD223, is expressed on multiple cell types including conventional T cells, NK cells, B cells [109], and unconventional T cells [109,110,111]. LAG-3 is an IgSF transmembrane protein with four extracellular domains that have similar folding patterns with CD4 in both humans and mice, indicating that LAG-3 can also bind to MHC-II, although at a different site than CD4 [112]. However, the intracellular regions of LAG-3 and CD4 do not have similarities [109,112]. Other LAG-3 ligands include FGL-1 (Fibrinogen-like Protein 1), Gal-3 (Galectin-3), and LSECtin (Lymph Node Sinusoidal Endothelial Cell C-type Lectin) and each has been shown to induce LAG-3-mediated inhibition of T cell activation [113,114,115], supporting the idea that LAG-3 could exert its inhibitory action independently of CD4. Unlike other ICRs, the intracellular region of LAG-3 lacks a typical cytoplasmic inhibitory ITIM or ITSM motif to inhibit T cell activation. Instead, the cytoplasmic domain of LAG-3 has three conserved regions in mice and humans that are not found in other ICRs [112,116]. Such regions include: an FSAL motif, a KIEELE motif, and an EX/EP repeat motif in the C-terminal region [109,116]. A study to identify the role of each motif in the inhibitory function of LAG-3, highlighted the importance of FSAL and dismissed the significance of KIEELE [117]. In particular, it was demonstrated that LAG-3 can transduce its inhibitory signals through the FSAL motif and EX/EP repeats by inhibition of IL-2 production [117]. Other in vitro and in vivo studies have demonstrated the importance of a lysine residue in KIEELE motif for the negative regulatory function of LAG-3 [118,119]. Furthermore, a LAG-3 associated protein (LAP) capable of interacting with the EX/EP motif has been identified [120]. Although it was proposed that LAP would be important in clustering LAG-3 into lipid rafts to promote signal transduction [120], there is not enough evidence in support of this hypothesis. Besides, it has been demonstrated that mutants lacking the EP motif are able to maintain LAG-3 activity [118], indicating that LAP may not be important for LAG-3 function. Although the available evidence suggests a discrepancy in the importance of the intracellular motifs of LAG-3, it is clear that LAG-3 inhibits immune cell activation through non-canonical inhibitory mechanisms compared to other ICRs. This suggests that the use of LAG-3 in immunotherapy combined with other ICRs would yield synergistic effects.

It has been reported that the inhibitory function of LAG-3 is correlated with its expression levels on the cell surface [117]. In a recent study, melanoma patients showed higher proportions of both circulating and tumor-infiltrating γδ T cells expressing LAG-3 compared to control groups, suggesting that LAG-3 may be crucial for immune escape and tumor progression by inhibition of γδ T cells [121]. Furthermore, the expression of LAG-3 in tumor-infiltrating γδ T cells was associated with earlier relapse and shorter overall survival [121]. More detailed studies on the putative role of LAG-3 in γδ T cells in the context of cancer are lacking. LAG-3 together with an assortment of exhaustion markers are highly expressed in γδ T cells derived from patients infected with *Plasmodium vivax* compared to uninfected controls [70]. In mice infected with *P. berghei* XAT, IFN-γ production by Vγ1^+^ γδ T cells was significantly reduced in the late phases of infection, which coincided with increased expression of LAG-3, as well as other ICRs [122].

Mass cytometry by time-of-flight (CyTOF) analysis from non–small cell lung cancer (NSCLC) patient samples showed that LAG-3 and PD-1 were mainly expressed in type I NKT and CD8^+^ T cells [123]. Consistent with this, the co-expression of these ICRs was associated with higher levels of activation markers, such as CD69, granzyme-B, and Ki-67, among others [123]. Besides, the authors suggested that LAG-3 expression could be considered for the selection of patients for immunotherapy since LAG-3 overexpression was negatively correlated with survival in patients with NSCLC that had been treated with PD-1 inhibitors, indicating that tumors in which immune evasion is mediated by LAG-3 are less sensitive to PD-1 blockade [123]. In this regard, early results in a clinical trial with LAG-3 inhibitors showed promising results in patients with advanced melanoma with resistance to PD-1 blockers [123,124]. Whether LAG-3-mediated regulation of NKT cells is of critical importance in cancer immunity remains to be elucidated. Similar to other cell types, chronic infection, such as HIV, results in elevated surface LAG-3 on type I NKT cells and reduced inhibition cytokine production [125]. Besides evidence that the exhausted phenotype of MAIT cells following exposure to bacterial antigens can be reversed by LAG-3 blockade [126], the role of LAG-3 in these cells in the context of cancer or inflammation is unknown.

## 10. TIM-3

T cell immunoglobulin and mucin-domain containing-3 (TIM-3) is an IgSF member expressed on the surface of T cells, B cells, NK cells, DCs, macrophages, and other immune cells [127,128,129,130]. TIM-3 can bind several ligands commonly found in the tumor microenvironment, including galectin-9 (Gal-9), HMGB-1, phosphatidylserine (PtdSer), and cell adhesion molecule 1 (Ceacam-1) [131,132,133,134]. The downstream signaling triggered by TIM-3 ligation is complex and is still being studied. In contrast to most inhibitory receptors, TIM-3 does not contain classical ITIM or ITSM motives [135]. In the absence of ligand, HLA- B-associated transcript 3 (Bat3) binds to the cytoplasmic tail of TIM-3, which results in the recruitment of the active form of Lck, known to promote TCR signaling [136,137]. The activation of TIM-3 by Gal-9 or Cecam-1, on the other hand, triggers the phosphorylation of conserved tyrosine residues on the cytoplasmic tail of this receptor [136]. As a result, Bat3 is released and SH2-domain containing kinases such as Fyn can be recruited in its place [138]. The interaction of Fyn with TIM-3 leads to the activation of PAG and CSK, which in turn phosphorylates an inhibitory residue on Lck, resulting in the inhibition of TCR signaling [136]. TIM-3 ligation has been shown to downregulate antitumor αβ T cell responses [131,135]. Thus, TIM-3-blocking agents are currently being tested in the clinic [131,135,139].

Recent studies suggest that TIM-3 also plays a role in the regulation of γδ T cell responses. This was first described in the infection field, where children exposed to *Plasmodium falciparum* exhibited higher TIM-3 expression in Vδ2 cells [140]. These TIM-3^+^ Vδ2 cells showed reduced proliferation and cytokine production following stimulation, which was associated with asymptomatic malaria infection [141]. More recently, Vδ2 cells isolated from acute myeloid leukemia (AML) and colorectal cancer patients displayed increased TIM-3 expression and a dysfunctional phenotype when compared to healthy controls [142,143,144]. Activation of TIM-3 with Gal-9 lowered Vδ2 cell cytotoxicity towards colon cancer cell lines by reducing production of perforin and granzyme B through the ERK1/2 pathway [144]. In addition, Vδ2 cells from AML patients showed impaired proliferative capacity upon IL-21 stimulation, which was restored by blocking TIM-3 signaling [142]. When both TIM-3 and PD-1 expression were investigated, Vδ2 cells that co-expressed TIM-3 and PD-1 exhibited the lowest production of IFN-γ and TNF-α compared to all other Vδ2 populations [143]. Interestingly, anti-TIM-3 or anti-TIM-3 plus anti-PD-1 blocking antibodies, but not anti-PD-1 alone, increased cytokine production [143], highlighting the importance of TIM-3 inhibition for functional restoration of γδ T cells.

In line with the above, blockade of TIM-3 signaling increased cytokine production by Vδ2 cells, but did not affect their proliferation [145]. The authors found that Vδ2 cells upregulated TIM-3 following TCR or TNF stimulation, and that TIM-3 ligation induced apoptosis through caspase-3, which was reversed by TIM-3 blockade [145]. Furthermore, in a murine model of breast cancer, combining adoptive transfer of γδ T cells and anti-TIM-3 antibodies enhanced anti-tumor responses compared to γδ T cell transfer alone [145]. The beneficial effects of γδ T cell transfer/anti-TIM-3 could be further improved by the addition of a bispecific anti-CD3/anti-EpCAM antibody, again showing the potential benefits of combination therapies involving TIM-3 inhibition [145]. Since several stimuli including not only TNF and TCR activation, but also IL-21 and anti-PD-1 administration have been reported to upregulate TIM-3 on Vδ2 cells [16,17], the previous study poses an interesting strategy in combining TIM-3 blockade with γδ T cell adoptive transfer protocols to prevent functional impairment of the transferred cells.

The role of TIM-3 on MAIT and NKT cells remains largely unknown. Elevated levels of TIM-3 have been reported on these cells during infection and in cancer patients, often in combination with other inhibitory receptors [76,104,106,126,146,147,148]. In addition, some studies show TIM-3 upregulation following MAIT or NKT cell activation [106,149,150,151]. There is, however, very limited evidence regarding the functionality of TIM-3 on these unconventional T cells. A study showed that following α-GalCer stimulation, murine hepatic NKT cells that express TIM-3 increase proliferation and produce higher levels of IFN-γ and IL-4 compared to their TIM-3^−^ counterpart [149]. In contrast, TIM-3^+^ NKT cells obtained from chronic hepatitis B patients, showed an impaired capacity to produce IFN-γ and IL-4 upon stimulation, which was partially reverted by TIM-3 or PD-1 blocking agents [150]. The ability of TIM-3/Gal-9 to induce apoptosis on NKT cells is currently unclear [149,152].

## 11. Concluding Remarks

It is evident from the above that our understanding of how ICRs regulate innate-like T cell responses is at its infancy. There are many fundamental questions that remain unanswered. Given their unconventional TCR interactions, how do ICRs inhibit γδ T, MAIT and NKT cells? Is innate activation by cytokines regulated by ICRs? Importantly, can we target innate-like T cells with CPI therapy in order to restore anti-cancer immunity, while at the same time subverting the tumor’s ability to evade MHC-restricted T cells? What are the chances that CPI therapy will over activate tumor-promoting IL-17-producing innate-like T cells? Are adverse effects associated with CPI therapy driven by unconventional T cells? We would like to propose (as shown in Figure 3) that elucidating the biological implications of ICR-mediated inhibition of unconventional T cells has the potential to unravel novel and important therapeutic avenues, particularly in the context of cancer immunotherapy.

## Figures and Tables

**Table 1:** ICRs with potentially important regulatory role in unconventional T cells. For each ICR, intracellular signaling domains, ligands, drugs in ongoing clinical trials, and drugs approved for use in cancer immunotherapy are shown. BsAb: bispecific antibody; all other abbreviations as in text.

Receptor	Signaling Domains	Ligands	Drugs in Clinical Trials	Approved Drugs
BTLA	ITIM/ITSM	HVEM	TAB004 JS004	
CTLA-4	Tyr-Val-Lys-Met	B7.1 B7.2	AGEN1884 AGEN1181 BMS-986218 IBI310 CS1002 KN044 MGD019 (BsAb PD-1/CTLA-4) SI-B003 (BsAb PD-1/CTLA-4) AK104 (BsAb PD-1/CTLA-4)	Ipilimumab
PD-1	ITIM/ITSM	PD-L1 PD-L2	Toripalimab BAT1306 SHR1210 Sym021 AGEN2034 JS001 CS1003	anti-PD-1: Nivolumab Pembrolizumab Cemiplimab anti-PD-L1: Avelumab Atezolizumab Durvalumab
LAG-3	FSAL motif KIEELE motif EX/EP repeats	MHC class II FGL-1 Gal-3 LSECtin	Relatlimab MGD013 (BsAb PD-1/LAG-3) LAG-525 REGN3767 IMP321 EMB-02 (BsAb PD-1/LAG-3) Sym022 BMS-986016 RO7247669 (BsAb PD-1/LAG-3) FS118 (BsAb LAG-3/PD-1) INCAGN02385 TSR-033 BMS-986213	
TIM-3	Tyr256 (Tyr265 in human) Tyr263	Gal-9 PtdSer Ceacam-1 HMGB-1	TSR-022 RO7121661 (BsAb PD-1/TIM-3) BGB-A425 Sym023 INCAGN02390 MGB453 LY3321367	

## References

[1] Godfrey D.I., Uldrich A.P., McCluskey J., Rossjohn J., Moody D.B. (2015). The burgeoning family of unconventional T cells. Nat. Immunol..

[2] Karunakaran M.M., Willcox C.R., Salim M., Paletta D., Fichtner A.S., Noll A., Starick L., Nohren A., Begley C.R., Berwick K.A. (2020). Butyrophilin-2A1 Directly Binds Germline-Encoded Regions of the Vgamma9Vdelta2 TCR and Is Essential for Phosphoantigen Sensing. Immunity.

[3] Le Nours J., Gherardin N.A., Ramarathinam S.H., Awad W., Wiede F., Gully B.S., Khandokar Y., Praveena T., Wubben J.M., Sandow J.J. (2019). A class of gammadelta T cell receptors recognize the underside of the antigen-presenting molecule MR1. Science.

[4] Willcox C.R., Vantourout P., Salim M., Zlatareva I., Melandri D., Zanardo L., George R., Kjaer S., Jeeves M., Mohammed F. (2019). Butyrophilin-like 3 Directly Binds a Human Vgamma4(+) T Cell Receptor Using a Modality Distinct from Clonally-Restricted Antigen. Immunity.

[5] Crosby C.M., Kronenberg M. (2018). Tissue-specific functions of invariant natural killer T cells. Nat. Rev. Immunol..

[6] Klenerman P., Hinks T.S.C., Ussher J.E. (2021). Biological functions of MAIT cells in tissues. Mol. Immunol..

[7] Ribot J.C., Lopes N., Silva-Santos B. (2020). Gammadelta T cells in tissue physiology and surveillance. Nat. Rev. Immunol..

[8] Edholm E.S., Banach M., Robert J. (2016). Evolution of innate-like T cells and their selection by MHC class I-like molecules. Immunogenetics.

[9] Hirano M., Guo P., McCurley N., Schorpp M., Das S., Boehm T., Cooper M.D. (2013). Evolutionary implications of a third lymphocyte lineage in lampreys. Nature.

[10] Strauss L., Mahmoud M.A.A., Weaver J.D., Tijaro-Ovalle N.M., Christofides A., Wang Q., Pal R., Yuan M., Asara J., Patsoukis N. (2020). Targeted deletion of PD-1 in myeloid cells induces antitumor immunity. Sci. Immunol..

[11] Gordon S.R., Maute R.L., Dulken B.W., Hutter G., George B.M., McCracken M.N., Gupta R., Tsai J.M., Sinha R., Corey D. (2017). PD-1 expression by tumour-associated macrophages inhibits phagocytosis and tumour immunity. Nature.

[12] Odorizzi P.M., Wherry E.J. (2012). Inhibitory receptors on lymphocytes: Insights from infections. J. Immunol..

[13] Fuertes Marraco S.A., Neubert N.J., Verdeil G., Speiser D.E. (2015). Inhibitory Receptors Beyond T Cell Exhaustion. Front. Immunol..

[14] De Sousa Linhares A., Leitner J., Grabmeier-Pfistershammer K., Steinberger P. (2018). Not All Immune Checkpoints Are Created Equal. Front. Immunol..

[15] Lorenz U. (2009). SHP-1 and SHP-2 in T cells: Two phosphatases functioning at many levels. Immunol. Rev..

[16] Wei S.C., Duffy C.R., Allison J.P. (2018). Fundamental Mechanisms of Immune Checkpoint Blockade Therapy. Cancer Discov..

[17] Sedy J.R., Gavrieli M., Potter K.G., Hurchla M.A., Lindsley R.C., Hildner K., Scheu S., Pfeffer K., Ware C.F., Murphy T.L. (2005). B and T lymphocyte attenuator regulates T cell activation through interaction with herpesvirus entry mediator. Nat. Immunol..

[18] Ward-Kavanagh L.K., Lin W.W., Sedy J.R., Ware C.F. (2016). The TNF Receptor Superfamily in Co-stimulating and Co-inhibitory Responses. Immunity.

[19] Sedy J., Bekiaris V., Ware C.F. (2015). Tumor necrosis factor superfamily in innate immunity and inflammation. Cold Spring Harb. Perspect. Biol..

[20] Cheung T.C., Oborne L.M., Steinberg M.W., Macauley M.G., Fukuyama S., Sanjo H., D’Souza C., Norris P.S., Pfeffer K., Murphy K.M. (2009). T cell intrinsic heterodimeric complexes between HVEM and BTLA determine receptivity to the surrounding microenvironment. J. Immunol..

[21] Petrelli A., Mijnheer G., Hoytema van Konijnenburg D.P., van der Wal M.M., Giovannone B., Mocholi E., Vazirpanah N., Broen J.C., Hijnen D., Oldenburg B. (2018). PD-1+CD8+ T cells are clonally expanding effectors in human chronic inflammation. J. Clin. Investig..

[22] Wherry E.J., Kurachi M. (2015). Molecular and cellular insights into T cell exhaustion. Nat. Rev. Immunol..

[23] Sharma P., Allison J.P. (2015). The future of immune checkpoint therapy. Science.

[24] Postow M.A., Callahan M.K., Wolchok J.D. (2015). Immune Checkpoint Blockade in Cancer Therapy. J. Clin. Oncol..

[25] Tundo G.R., Sbardella D., Lacal P.M., Graziani G., Marini S. (2019). On the Horizon: Targeting Next-Generation Immune Checkpoints for Cancer Treatment. Chemotherapy.

[26] Rotte A., Jin J.Y., Lemaire V. (2018). Mechanistic overview of immune checkpoints to support the rational design of their combinations in cancer immunotherapy. Ann. Oncol..

[27] Jenkins R.W., Barbie D.A., Flaherty K.T. (2018). Mechanisms of resistance to immune checkpoint inhibitors. Br. J. Cancer.

[28] Sharma P., Hu-Lieskovan S., Wargo J.A., Ribas A. (2017). Primary, Adaptive, and Acquired Resistance to Cancer Immunotherapy. Cell.

[29] Ceci C., Atzori M.G., Lacal P.M., Graziani G. (2020). Targeting Tumor-Associated Macrophages to Increase the Efficacy of Immune Checkpoint Inhibitors: A Glimpse into Novel Therapeutic Approaches for Metastatic Melanoma. Cancers.

[30] Bekiaris V., Sedy J.R., Macauley M.G., Rhode-Kurnow A., Ware C.F. (2013). The Inhibitory Receptor BTLA Controls gammadelta T Cell Homeostasis and Inflammatory Responses. Immunity.

[31] Imai Y., Ayithan N., Wu X., Yuan Y., Wang L., Hwang S.T. (2015). Cutting Edge: PD-1 Regulates Imiquimod-Induced Psoriasiform Dermatitis through Inhibition of IL-17A Expression by Innate γδ-Low T Cells. J. Immunol..

[32] Kadekar D., Agerholm R., Vinals M.T., Rizk J., Bekiaris V. (2020). The immune checkpoint receptor associated phosphatases SHP-1 and SHP-2 are not required for gammadeltaT17 cell development, activation, or skin inflammation. Eur. J. Immunol..

[33] Martins F., Sofiya L., Sykiotis G.P., Lamine F., Maillard M., Fraga M., Shabafrouz K., Ribi C., Cairoli A., Guex-Crosier Y. (2019). Adverse effects of immune-checkpoint inhibitors: Epidemiology, management and surveillance. Nat. Rev. Clin. Oncol..

[34] Haas J.D., Ravens S., Duber S., Sandrock I., Oberdorfer L., Kashani E., Chennupati V., Fohse L., Naumann R., Weiss S. (2012). Development of interleukin-17-producing gammadelta T cells is restricted to a functional embryonic wave. Immunity.

[35] Tan L., Fichtner A.S., Bruni E., Odak I., Sandrock I., Bubke A., Borchers A., Schultze-Florey C., Koenecke C., Forster R. (2021). A fetal wave of human type 3 effector gammadelta cells with restricted TCR diversity persists into adulthood. Sci Immunol.

[36] Ribot J.C., de Barros A., Pang D.J., Neves J.F., Peperzak V., Roberts S.J., Girardi M., Borst J., Hayday A.C., Pennington D.J. (2009). CD27 is a thymic determinant of the balance between interferon-gamma- and interleukin 17-producing gammadelta T cell subsets. Nat. Immunol..

[37] Turchinovich G., Hayday A.C. (2011). Skint-1 identifies a common molecular mechanism for the development of interferon-gamma-secreting versus interleukin-17-secreting gammadelta T cells. Immunity.

[38] Wencker M., Turchinovich G., Di Marco Barros R., Deban L., Jandke A., Cope A., Hayday A.C. (2014). Innate-like T cells straddle innate and adaptive immunity by altering antigen-receptor responsiveness. Nat. Immunol..

[39] Munoz-Ruiz M., Ribot J.C., Grosso A.R., Goncalves-Sousa N., Pamplona A., Pennington D.J., Regueiro J.R., Fernandez-Malave E., Silva-Santos B. (2016). TCR signal strength controls thymic differentiation of discrete proinflammatory gammadelta T cell subsets. Nat. Immunol..

[40] Sumaria N., Grandjean C.L., Silva-Santos B., Pennington D.J. (2017). Strong TCRgammadelta Signaling Prohibits Thymic Development of IL-17A-Secreting gammadelta T Cells. Cell Rep..

[41] Papotto P.H., Ribot J.C., Silva-Santos B. (2017). IL-17+ gammadelta T cells as kick-starters of inflammation. Nat. Immunol..

[42] Di Marco Barros R., Roberts N.A., Dart R.J., Vantourout P., Jandke A., Nussbaumer O., Deban L., Cipolat S., Hart R., Iannitto M.L. (2016). Epithelia Use Butyrophilin-like Molecules to Shape Organ-Specific gammadelta T Cell Compartments. Cell.

[43] Rigau M., Ostrouska S., Fulford T.S., Johnson D.N., Woods K., Ruan Z., McWilliam H.E.G., Hudson C., Tutuka C., Wheatley A.K. (2020). Butyrophilin 2A1 is essential for phosphoantigen reactivity by gammadelta T cells. Science.

[44] Marlin R., Pappalardo A., Kaminski H., Willcox C.R., Pitard V., Netzer S., Khairallah C., Lomenech A.M., Harly C., Bonneville M. (2017). Sensing of cell stress by human gammadelta TCR-dependent recognition of annexin A2. Proc. Natl. Acad. Sci. USA.

[45] Alexander A.A., Maniar A., Cummings J.S., Hebbeler A.M., Schulze D.H., Gastman B.R., Pauza C.D., Strome S.E., Chapoval A.I. (2008). Isopentenyl pyrophosphate-activated CD56+ γδ T lymphocytes display potent antitumor activity toward human squamous cell carcinoma. Clin. Cancer Res..

[46] Blazquez J.L., Benyamine A., Pasero C., Olive D. (2018). New Insights Into the Regulation of gammadelta T Cells by BTN3A and Other BTN/BTNL in Tumor Immunity. Front. Immunol..

[47] Zhao Y., Niu C., Cui J. (2018). Gamma-delta (gammadelta) T cells: Friend or foe in cancer development?. J. Transl. Med..

[48] Kobayashi H., Tanaka Y. (2015). gammadelta T Cell Immunotherapy—A Review. Pharmaceuticals.

[49] Almeida A.R., Correia D.V., Fernandes-Platzgummer A., da Silva C.L., da Silva M.G., Anjos D.R., Silva-Santos B. (2016). Delta One T Cells for Immunotherapy of Chronic Lymphocytic Leukemia: Clinical-Grade Expansion/Differentiation and Preclinical Proof of Concept. Clin. Cancer Res..

[50] Di Lorenzo B., Simoes A.E., Caiado F., Tieppo P., Correia D.V., Carvalho T., da Silva M.G., Dechanet-Merville J., Schumacher T.N., Prinz I. (2019). Broad Cytotoxic Targeting of Acute Myeloid Leukemia by Polyclonal Delta One T Cells. Cancer Immunol. Res..

[51] Sebestyen Z., Prinz I., Dechanet-Merville J., Silva-Santos B., Kuball J. (2019). Translating gammadelta (gammadelta) T cells and their receptors into cancer cell therapies. Nat. Rev. Drug Discov..

[52] Lalor S.J., McLoughlin R.M. (2016). Memory gammadelta T Cells-Newly Appreciated Protagonists in Infection and Immunity. Trends Immunol..

[53] Comeau K., Paradis P., Schiffrin E.L. (2020). Human and murine memory gammadelta T cells: Evidence for acquired immune memory in bacterial and viral infections and autoimmunity. Cell Immunol..

[54] Godfrey D.I., Le Nours J., Andrews D.M., Uldrich A.P., Rossjohn J. (2018). Unconventional T Cell Targets for Cancer Immunotherapy. Immunity.

[55] Cogswell D.T., Gapin L., Tobin H.M., McCarter M.D., Tobin R.P. (2021). MAIT Cells: Partners or Enemies in Cancer Immunotherapy?. Cancers.

[56] O’Neill C., Cassidy F.C., O’Shea D., Hogan A.E. (2021). Mucosal Associated Invariant T Cells in Cancer-Friend or Foe?. Cancers.

[57] Sedy J.R., Ramezani-Rad P. (2019). HVEM network signaling in cancer. Adv. Cancer Res..

[58] Boice M., Salloum D., Mourcin F., Sanghvi V., Amin R., Oricchio E., Jiang M., Mottok A., Denis-Lagache N., Ciriello G. (2016). Loss of the HVEM Tumor Suppressor in Lymphoma and Restoration by Modified CAR-T Cells. Cell.

[59] Gertner-Dardenne J., Fauriat C., Orlanducci F., Thibult M.L., Pastor S., Fitzgibbon J., Bouabdallah R., Xerri L., Olive D. (2013). The co-receptor BTLA negatively regulates human Vgamma9Vdelta2 T-cell proliferation: A potential way of immune escape for lymphoma cells. Blood.

[60] Hwang H.J., Lee J.J., Kang S.H., Suh J.K., Choi E.S., Jang S., Hwang S.H., Koh K.N., Im H.J., Kim N. (2021). The BTLA and PD-1 signaling pathways independently regulate the proliferation and cytotoxicity of human peripheral blood gammadelta T cells. Immun. Inflamm. Dis..

[61] Iwata A., Watanabe N., Oya Y., Owada T., Ikeda K., Suto A., Kagami S., Hirose K., Kanari H., Kawashima S. (2010). Protective roles of B and T lymphocyte attenuator in NKT cell-mediated experimental hepatitis. J. Immunol..

[62] Miller M.L., Sun Y., Fu Y.X. (2009). Cutting edge: B and T lymphocyte attenuator signaling on NKT cells inhibits cytokine release and tissue injury in early immune responses. J. Immunol..

[63] Sekar D., Govene L., Del Rio M.L., Sirait-Fischer E., Fink A.F., Brune B., Rodriguez-Barbosa J.I., Weigert A. (2018). Downregulation of BTLA on NKT Cells Promotes Tumor Immune Control in a Mouse Model of Mammary Carcinoma. Int. J. Mol. Sci..

[64] Krummel M.F., Allison J.P. (1995). CD28 and CTLA-4 have opposing effects on the response of T cells to stimulation. J. Exp. Med..

[65] Walunas T.L., Lenschow D.J., Bakker C.Y., Linsley P.S., Freeman G.J., Green J.M., Thompson C.B., Bluestone J.A. (1994). CTLA-4 can function as a negative regulator of T cell activation. Immunity.

[66] Waterhouse P., Penninger J.M., Timms E., Wakeham A., Shahinian A., Lee K.P., Thompson C.B., Griesser H., Mak T.W. (1995). Lymphoproliferative disorders with early lethality in mice deficient in Ctla-4. Science.

[67] Tivol E.A., Borriello F., Schweitzer A.N., Lynch W.P., Bluestone J.A., Sharpe A.H. (1995). Loss of CTLA-4 leads to massive lymphoproliferation and fatal multiorgan tissue destruction, revealing a critical negative regulatory role of CTLA-4. Immunity.

[68] Chambers C.A., Sullivan T.J., Allison J.P. (1997). Lymphoproliferation in CTLA-4-deficient mice is mediated by costimulation-dependent activation of CD4+ T cells. Immunity.

[69] Leach D.R., Krummel M.F., Allison J.P. (1996). Enhancement of antitumor immunity by CTLA-4 blockade. Science.

[70] Gogoi D., Biswas D., Borkakoty B., Mahanta J. (2018). Exposure to Plasmodium vivax is associated with the increased expression of exhaustion markers on γδ T lymphocytes. Parasite Immunol..

[71] Cimini E., Viola D., Cabeza-Cabrerizo M., Romanelli A., Tumino N., Sacchi A., Bordoni V., Casetti R., Turchi F., Martini F. (2017). Different features of Vdelta2 T and NK cells in fatal and non-fatal human Ebola infections. PLoS Negl. Trop. Dis..

[72] Wistuba-Hamprecht K., Martens A., Haehnel K., Geukes Foppen M., Yuan J., Postow M.A., Wong P., Romano E., Khammari A., Dreno B. (2016). Proportions of blood-borne Vdelta1+ and Vdelta2+ T-cells are associated with overall survival of melanoma patients treated with ipilimumab. Eur. J. Cancer.

[73] Peters C., Oberg H.H., Kabelitz D., Wesch D. (2014). Phenotype and regulation of immunosuppressive Vdelta2-expressing gammadelta T cells. Cell Mol. Life Sci..

[74] Li J., Zhu H., Wang S., Ye P., Liu C., Wu J., Xia J. (2012). Blockade of NKG2D synergized with CTLA4-Ig in promoting long-term graft survival in murine models of cardiac transplantation. Transplantation.

[75] Bottcher K., Rombouts K., Saffioti F., Roccarina D., Rosselli M., Hall A., Luong T., Tsochatzis E.A., Thorburn D., Pinzani M. (2018). MAIT cells are chronically activated in patients with autoimmune liver disease and promote profibrogenic hepatic stellate cell activation. Hepatology.

[76] Yong Y.K., Saeidi A., Tan H.Y., Rosmawati M., Enström P.F., Batran R.A., Vasuki V., Chattopadhyay I., Murugesan A., Vignesh R. (2018). Hyper-Expression of PD-1 Is Associated with the Levels of Exhausted and Dysfunctional Phenotypes of Circulating CD161(++)TCR iVα7.2(+) Mucosal-Associated Invariant T Cells in Chronic Hepatitis B Virus Infection. Front. Immunol..

[77] Berkson J.D., Slichter C.K., DeBerg H.A., Delaney M.A., Woodward-Davis A.S., Maurice N.J., Lwo Y., Ko A., Hsu J., Chiu Y.W. (2020). Inflammatory Cytokines Induce Sustained CTLA-4 Cell Surface Expression on Human MAIT Cells. Immunohorizons.

[78] Ishida Y., Agata Y., Shibahara K., Honjo T. (1992). Induced expression of PD-1, a novel member of the immunoglobulin gene superfamily, upon programmed cell death. EMBO J..

[79] Iwai Y., Ishida M., Tanaka Y., Okazaki T., Honjo T., Minato N. (2002). Involvement of PD-L1 on tumor cells in the escape from host immune system and tumor immunotherapy by PD-L1 blockade. Proc. Natl. Acad. Sci. USA.

[80] Keir M.E., Butte M.J., Freeman G.J., Sharpe A.H. (2008). PD-1 and its ligands in tolerance and immunity. Annu. Rev. Immunol..

[81] Okazaki T., Maeda A., Nishimura H., Kurosaki T., Honjo T. (2001). PD-1 immunoreceptor inhibits B cell receptor-mediated signaling by recruiting src homology 2-domain-containing tyrosine phosphatase 2 to phosphotyrosine. Proc. Natl. Acad. Sci. USA.

[82] Chemnitz J.M., Parry R.V., Nichols K.E., June C.H., Riley J.L. (2004). SHP-1 and SHP-2 associate with immunoreceptor tyrosine-based switch motif of programmed death 1 upon primary human T cell stimulation, but only receptor ligation prevents T cell activation. J. Immunol..

[83] Iwasaki M., Tanaka Y., Kobayashi H., Murata-Hirai K., Miyabe H., Sugie T., Toi M., Minato N. (2011). Expression and function of PD-1 in human γδ T cells that recognize phosphoantigens. Eur. J. Immunol..

[84] Hoeres T., Holzmann E., Smetak M., Birkmann J., Wilhelm M. (2019). PD-1 signaling modulates interferon-γ production by Gamma Delta (γδ) T-Cells in response to leukemia. Oncoimmunology.

[85] Hsu H., Boudova S., Mvula G., Divala T.H., Mungwira R.G., Harman C., Laufer M.K., Pauza C.D., Cairo C. (2016). Prolonged PD1 Expression on Neonatal Vδ2 Lymphocytes Dampens Proinflammatory Responses: Role of Epigenetic Regulation. J. Immunol..

[86] Dondero A., Pastorino F., Della Chiesa M., Corrias M.V., Morandi F., Pistoia V., Olive D., Bellora F., Locatelli F., Castellano A. (2016). PD-L1 expression in metastatic neuroblastoma as an additional mechanism for limiting immune surveillance. Oncoimmunology.

[87] Hu G., Wu P., Cheng P., Zhang Z., Wang Z., Yu X., Shao X., Wu D., Ye J., Zhang T. (2017). Tumor-infiltrating CD39(+)γδTregs are novel immunosuppressive T cells in human colorectal cancer. Oncoimmunology.

[88] Castella B., Foglietta M., Sciancalepore P., Rigoni M., Coscia M., Griggio V., Vitale C., Ferracini R., Saraci E., Omedé P. (2015). Anergic bone marrow Vγ9Vδ2 T cells as early and long-lasting markers of PD-1-targetable microenvironment-induced immune suppression in human myeloma. Oncoimmunology.

[89] Rossi C., Gravelle P., Decaup E., Bordenave J., Poupot M., Tosolini M., Franchini D.M., Laurent C., Morin R., Lagarde J.M. (2019). Boosting γδ T cell-mediated antibody-dependent cellular cytotoxicity by PD-1 blockade in follicular lymphoma. Oncoimmunology.

[90] Xiong D., Wang Y., You M. (2020). A gene expression signature of TREM2(hi) macrophages and γδ T cells predicts immunotherapy response. Nat. Commun..

[91] Kim J.H., Choi Y.J., Lee B.H., Song M.Y., Ban C.Y., Kim J., Park J., Kim S.E., Kim T.G., Park S.H. (2016). Programmed cell death ligand 1 alleviates psoriatic inflammation by suppressing IL-17A production from programmed cell death 1-high T cells. J. Allergy Clin. Immunol..

[92] Sheng Y., Chen K., Jiang W., Wu Z., Zhang W., Jing H., Wang L., Qu C., Ren H. (2021). PD-1 restrains IL-17A production from γδ T cells to modulate acute radiation-induced lung injury. Transl. Lung Cancer Res..

[93] Chang W.S., Kim J.Y., Kim Y.J., Kim Y.S., Lee J.M., Azuma M., Yagita H., Kang C.Y. (2008). Cutting edge: Programmed death-1/programmed death ligand 1 interaction regulates the induction and maintenance of invariant NKT cell anergy. J. Immunol..

[94] Kamata T., Suzuki A., Mise N., Ihara F., Takami M., Makita Y., Horinaka A., Harada K., Kunii N., Yoshida S. (2016). Blockade of programmed death-1/programmed death ligand pathway enhances the antitumor immunity of human invariant natural killer T cells. Cancer Immunol. Immunother..

[95] Parekh V.V., Lalani S., Kim S., Halder R., Azuma M., Yagita H., Kumar V., Wu L., Kaer L.V. (2009). PD-1/PD-L blockade prevents anergy induction and enhances the anti-tumor activities of glycolipid-activated invariant NKT cells. J. Immunol..

[96] Wang J., Cheng L., Wondimu Z., Swain M., Santamaria P., Yang Y. (2009). Cutting edge: CD28 engagement releases antigen-activated invariant NKT cells from the inhibitory effects of PD-1. J. Immunol..

[97] Durgan K., Ali M., Warner P., Latchman Y.E. (2011). Targeting NKT cells and PD-L1 pathway results in augmented anti-tumor responses in a melanoma model. Cancer Immunol. Immunother..

[98] Iyoda T., Ushida M., Kimura Y., Minamino K., Hayuka A., Yokohata S., Ehara H., Inaba K. (2010). Invariant NKT cell anergy is induced by a strong TCR-mediated signal plus co-stimulation. Int. Immunol..

[99] Wang X.F., Lei Y., Chen M., Chen C.B., Ren H., Shi T.D. (2013). PD-1/PDL1 and CD28/CD80 pathways modulate natural killer T cell function to inhibit hepatitis B virus replication. J. Viral Hepat..

[100] Wang Y., Bhave M.S., Yagita H., Cardell S.L. (2020). Natural Killer T-Cell Agonist α-Galactosylceramide and PD-1 Blockade Synergize to Reduce Tumor Development in a Preclinical Model of Colon Cancer. Front. Immunol..

[101] Kee S.J., Kwon Y.S., Park Y.W., Cho Y.N., Lee S.J., Kim T.J., Lee S.S., Jang H.C., Shin M.G., Shin J.H. (2012). Dysfunction of natural killer T cells in patients with active Mycobacterium tuberculosis infection. Infect. Immun..

[102] Jiang J., Wang X., An H., Yang B., Cao Z., Liu Y., Su J., Zhai F., Wang R., Zhang G. (2014). Mucosal-associated invariant T-cell function is modulated by programmed death-1 signaling in patients with active tuberculosis. Am. J. Respir. Crit. Care Med..

[103] Jiang J., Cao Z., Qu J., Liu H., Han H., Cheng X. (2020). PD-1-expressing MAIT cells from patients with tuberculosis exhibit elevated production of CXCL13. Scand. J. Immunol..

[104] Duan M., Goswami S., Shi J.Y., Wu L.J., Wang X.Y., Ma J.Q., Zhang Z., Shi Y., Ma L.J., Zhang S. (2019). Activated and Exhausted MAIT Cells Foster Disease Progression and Indicate Poor Outcome in Hepatocellular Carcinoma. Clin. Cancer Res..

[105] Melo A.M., O’Brien A.M., Phelan J.J., Kennedy S.A., Wood N.A.W., Veerapen N., Besra G.S., Clarke N.E., Foley E.K., Ravi A. (2019). Mucosal-Associated Invariant T Cells Display Diminished Effector Capacity in Oesophageal Adenocarcinoma. Front. Immunol..

[106] Rodin W., Sundström P., Ahlmanner F., Szeponik L., Zajt K.K., Wettergren Y., Bexe Lindskog E., Quiding Järbrink M. (2021). Exhaustion in tumor-infiltrating Mucosal-Associated Invariant T (MAIT) cells from colon cancer patients. Cancer Immunol. Immunother..

[107] Yao T., Shooshtari P., Haeryfar S.M.M. (2020). Leveraging Public Single-Cell and Bulk Transcriptomic Datasets to Delineate MAIT Cell Roles and Phenotypic Characteristics in Human Malignancies. Front. Immunol..

[108] De Biasi S., Gibellini L., Lo Tartaro D., Puccio S., Rabacchi C., Mazza E.M.C., Brummelman J., Williams B., Kaihara K., Forcato M. (2021). Circulating mucosal-associated invariant T cells identify patients responding to anti-PD-1 therapy. Nat. Commun..

[109] Graydon C.G., Mohideen S., Fowke K.R. (2020). LAG3′s Enigmatic Mechanism of Action. Front. Immunol..

[110] Workman C.J., Rice D.S., Dugger K.J., Kurschner C., Vignali D.A. (2002). Phenotypic analysis of the murine CD4-related glycoprotein, CD223 (LAG-3). Eur. J. Immunol..

[111] Byun H.J., Jung W.W., Lee D.S., Kim S., Kim S.J., Park C.G., Chung H.Y., Chun T. (2007). Proliferation of activated CD1d-restricted NKT cells is down-modulated by lymphocyte activation gene-3 signaling via cell cycle arrest in S phase. Cell Biol. Int..

[112] Andrews L.P., Marciscano A.E., Drake C.G., Vignali D.A. (2017). LAG3 (CD223) as a cancer immunotherapy target. Immunol. Rev..

[113] Wang J., Sanmamed M.F., Datar I., Su T.T., Ji L., Sun J., Chen L., Chen Y., Zhu G., Yin W. (2019). Fibrinogen-like Protein 1 Is a Major Immune Inhibitory Ligand of LAG-3. Cell.

[114] Kouo T., Huang L., Pucsek A.B., Cao M., Solt S., Armstrong T., Jaffee E. (2015). Galectin-3 Shapes Antitumor Immune Responses by Suppressing CD8+ T Cells via LAG-3 and Inhibiting Expansion of Plasmacytoid Dendritic Cells. Cancer Immunol. Res..

[115] Xu F., Liu J., Liu D., Liu B., Wang M., Hu Z., Du X., Tang L., He F. (2014). LSECtin expressed on melanoma cells promotes tumor progression by inhibiting antitumor T-cell responses. Cancer Res..

[116] Maruhashi T., Sugiura D., Okazaki I.M., Okazaki T. (2020). LAG-3: From molecular functions to clinical applications. J. Immunother. Cancer.

[117] Maeda T.K., Sugiura D., Okazaki I.M., Maruhashi T., Okazaki T. (2019). Atypical motifs in the cytoplasmic region of the inhibitory immune co-receptor LAG-3 inhibit T cell activation. J. Biol. Chem..

[118] Workman C.J., Dugger K.J., Vignali D.A. (2002). Cutting edge: Molecular analysis of the negative regulatory function of lymphocyte activation gene-3. J. Immunol..

[119] Workman C.J., Vignali D.A. (2003). The CD4-related molecule, LAG-3 (CD223), regulates the expansion of activated T cells. Eur. J. Immunol..

[120] Iouzalen N., Andreae S., Hannier S., Triebel F. (2001). LAP, a lymphocyte activation gene-3 (LAG-3)-associated protein that binds to a repeated EP motif in the intracellular region of LAG-3, may participate in the down-regulation of the CD3/TCR activation pathway. Eur. J. Immunol..

[121] Girard P., Charles J., Cluzel C., Degeorges E., Manches O., Plumas J., De Fraipont F., Leccia M.T., Mouret S., Chaperot L. (2019). The features of circulating and tumor-infiltrating γδ T cells in melanoma patients display critical perturbations with prognostic impact on clinical outcome. Oncoimmunology.

[122] Inoue S.I., Niikura M., Asahi H., Iwakura Y., Kawakami Y., Kobayashi F. (2017). Preferentially expanding Vγ1(+) γδ T cells are associated with protective immunity against Plasmodium infection in mice. Eur. J. Immunol..

[123] Datar I., Sanmamed M.F., Wang J., Henick B.S., Choi J., Badri T., Dong W., Mani N., Toki M., Mejías L.D. (2019). Expression Analysis and Significance of PD-1, LAG-3, and TIM-3 in Human Non-Small Cell Lung Cancer Using Spatially Resolved and Multiparametric Single-Cell Analysis. Clin. Cancer Res..

[124] Kwiatkowska D., Kluska P., Reich A. (2019). Beyond PD-1 Immunotherapy in Malignant Melanoma. Dermatol. Ther..

[125] Juno J.A., Stalker A.T., Waruk J.L., Oyugi J., Kimani M., Plummer F.A., Kimani J., Fowke K.R. (2015). Elevated expression of LAG-3, but not PD-1, is associated with impaired iNKT cytokine production during chronic HIV-1 infection and treatment. Retrovirology.

[126] Shaler C.R., Choi J., Rudak P.T., Memarnejadian A., Szabo P.A., Tun-Abraham M.E., Rossjohn J., Corbett A.J., McCluskey J., McCormick J.K. (2017). MAIT cells launch a rapid, robust and distinct hyperinflammatory response to bacterial superantigens and quickly acquire an anergic phenotype that impedes their cognate antimicrobial function: Defining a novel mechanism of superantigen-induced immunopathology and immunosuppression. PLoS Biol..

[127] Monney L., Sabatos C.A., Gaglia J.L., Ryu A., Waldner H., Chernova T., Manning S., Greenfield E.A., Coyle A.J., Sobel R.A. (2002). Th1-specific cell surface protein Tim-3 regulates macrophage activation and severity of an autoimmune disease. Nature.

[128] Gao X., Zhu Y., Li G., Huang H., Zhang G., Wang F., Sun J., Yang Q., Zhang X., Lu B. (2012). TIM-3 expression characterizes regulatory T cells in tumor tissues and is associated with lung cancer progression. PLoS ONE.

[129] Ndhlovu L.C., Lopez-Vergès S., Barbour J.D., Jones R.B., Jha A.R., Long B.R., Schoeffler E.C., Fujita T., Nixon D.F., Lanier L.L. (2012). Tim-3 marks human natural killer cell maturation and suppresses cell-mediated cytotoxicity. Blood.

[130] Nakayama M., Akiba H., Takeda K., Kojima Y., Hashiguchi M., Azuma M., Yagita H., Okumura K. (2009). Tim-3 mediates phagocytosis of apoptotic cells and cross-presentation. Blood.

[131] Kang C.W., Dutta A., Chang L.Y., Mahalingam J., Lin Y.C., Chiang J.M., Hsu C.Y., Huang C.T., Su W.T., Chu Y.Y. (2015). Apoptosis of tumor infiltrating effector TIM-3+CD8+ T cells in colon cancer. Sci. Rep..

[132] Chiba S., Baghdadi M., Akiba H., Yoshiyama H., Kinoshita I., Dosaka-Akita H., Fujioka Y., Ohba Y., Gorman J.V., Colgan J.D. (2012). Tumor-infiltrating DCs suppress nucleic acid-mediated innate immune responses through interactions between the receptor TIM-3 and the alarmin HMGB1. Nat. Immunol..

[133] Tan S., Xu Y., Wang Z., Wang T., Du X., Song X., Guo X., Peng J., Zhang J., Liang Y. (2020). Tim-3 Hampers Tumor Surveillance of Liver-Resident and Conventional NK Cells by Disrupting PI3K Signaling. Cancer Res..

[134] Huang Y.H., Zhu C., Kondo Y., Anderson A.C., Gandhi A., Russell A., Dougan S.K., Petersen B.S., Melum E., Pertel T. (2015). CEACAM1 regulates TIM-3-mediated tolerance and exhaustion. Nature.

[135] Das M., Zhu C., Kuchroo V.K. (2017). Tim-3 and its role in regulating anti-tumor immunity. Immunol. Rev..

[136] Wolf Y., Anderson A.C., Kuchroo V.K. (2020). TIM3 comes of age as an inhibitory receptor. Nat. Rev. Immunol..

[137] Rangachari M., Zhu C., Sakuishi K., Xiao S., Karman J., Chen A., Angin M., Wakeham A., Greenfield E.A., Sobel R.A. (2012). Bat3 promotes T cell responses and autoimmunity by repressing Tim-3–mediated cell death and exhaustion. Nat. Med..

[138] Lee J., Su E.W., Zhu C., Hainline S., Phuah J., Moroco J.A., Smithgall T.E., Kuchroo V.K., Kane L.P. (2011). Phosphotyrosine-dependent coupling of Tim-3 to T-cell receptor signaling pathways. Mol. Cell Biol..

[139] Lee J.B., Ha S.J., Kim H.R. (2021). Clinical Insights Into Novel Immune Checkpoint Inhibitors. Front Pharm..

[140] Jagannathan P., Kim C.C., Greenhouse B., Nankya F., Bowen K., Eccles-James I., Muhindo M.K., Arinaitwe E., Tappero J.W., Kamya M.R. (2014). Loss and dysfunction of Vδ2^+^ γδ T cells are associated with clinical tolerance to malaria. Sci. Transl. Med..

[141] Jagannathan P., Lutwama F., Boyle M.J., Nankya F., Farrington L.A., McIntyre T.I., Bowen K., Naluwu K., Nalubega M., Musinguzi K. (2017). Vδ2+ T cell response to malaria correlates with protection from infection but is attenuated with repeated exposure. Sci. Rep..

[142] Wu K., Zhao H., Xiu Y., Li Z., Zhao J., Xie S., Zeng H., Zhang H., Yu L., Xu B. (2019). IL-21-mediated expansion of Vγ9Vδ2 T cells is limited by the Tim-3 pathway. Int. Immunopharmacol..

[143] Wu K., Feng J., Xiu Y., Li Z., Lin Z., Zhao H., Zeng H., Xia W., Yu L., Xu B. (2020). Vδ2 T cell subsets, defined by PD-1 and TIM-3 expression, present varied cytokine responses in acute myeloid leukemia patients. Int. Immunopharmacol..

[144] Li X., Lu H., Gu Y., Zhang X., Zhang G., Shi T., Chen W. (2020). Tim-3 suppresses the killing effect of Vγ9Vδ2 T cells on colon cancer cells by reducing perforin and granzyme B expression. Exp. Cell Res..

[145] Guo Q., Zhao P., Zhang Z., Zhang J., Zhang Z., Hua Y., Han B., Li N., Zhao X., Hou L. (2020). TIM-3 blockade combined with bispecific antibody MT110 enhances the anti-tumor effect of γδ T cells. Cancer Immunol. Immunother..

[146] Kang S.J., Jin H.M., Cho Y.N., Oh T.H., Kim S.E., Kim U.J., Park K.H., Jang H.C., Jung S.I., Kee S.J. (2018). Dysfunction of Circulating Natural Killer T Cells in Patients With Scrub Typhus. J. Infect. Dis..

[147] Almeida J.S., Couceiro P., López-Sejas N., Alves V., Růžičková L., Tarazona R., Solana R., Freitas-Tavares P., Santos-Rosa M., Rodrigues-Santos P. (2019). NKT-Like (CD3+CD56+) Cells in Chronic Myeloid Leukemia Patients Treated With Tyrosine Kinase Inhibitors. Front. Immunol..

[148] Xu L.Y., Chen D.D., He J.Y., Lu C.C., Liu X.G., Le H.B., Wang C.Y., Zhang Y.K. (2014). Tim-3 expression by peripheral natural killer cells and natural killer T cells increases in patients with lung cancer—Reduction after surgical resection. Asian Pac. J. Cancer Prev..

[149] Tang Z.H., Liang S., Potter J., Jiang X., Mao H.Q., Li Z. (2013). Tim-3/galectin-9 regulate the homeostasis of hepatic NKT cells in a murine model of nonalcoholic fatty liver disease. J. Immunol..

[150] Yang Z., Lei Y., Chen C., Ren H., Shi T. (2015). Roles of the programmed cell death 1, T cell immunoglobulin mucin-3, and cluster of differentiation 288 pathways in the low reactivity of invariant natural killer T cells after chronic hepatitis B virus infection. Arch. Virol..

[151] Yao Y., Deng H., Li P., Zhang J., Zhang J., Wang D., Li S., Luo Y., Wei Z., Bi G. (2017). α-Lactose Improves the Survival of Septic Mice by Blockade of TIM-3 Signaling to Prevent NKT Cell Apoptosis and Attenuate Cytokine Storm. Shock.

[152] Kadowaki T., Morishita A., Niki T., Hara J., Sato M., Tani J., Miyoshi H., Yoneyama H., Masaki T., Hattori T. (2013). Galectin-9 prolongs the survival of septic mice by expanding Tim-3-expressing natural killer T cells and PDCA-1+ CD11c+ macrophages. Crit. Care.

